# The Emergence of Universal Immune Receptor T Cell Therapy for Cancer

**DOI:** 10.3389/fonc.2019.00176

**Published:** 2019-03-26

**Authors:** Nicholas G. Minutolo, Erin E. Hollander, Daniel J. Powell

**Affiliations:** ^1^Department of Pathology and Laboratory Medicine, Abramson Cancer Center, Perelman School of Medicine, University of Pennsylvania, Philadelphia, PA, United States; ^2^Department of Systems Pharmacology and Translational Therapeutics, University of Pennsylvania School of Medicine, Philadelphia, PA, United States; ^3^Pharmacology Graduate Group, University of Pennsylvania, Philadelphia, PA, United States; ^4^Center for Cellular Immunotherapies, University of Pennsylvania School of Medicine, Philadelphia, PA, United States; ^5^Department of Cancer Biology, University of Pennsylvania School of Medicine, Philadelphia, PA, United States

**Keywords:** chimeric antigen receptor (CAR), universal immune receptor, switchable CAR, cancer immunotherapy, T cell therapy

## Abstract

Chimeric antigen receptor (CAR) T cells have shown great success in the treatment of CD19+ hematological malignancies, leading to their recent approval by the FDA as a new cancer treatment modality. However, their broad use is limited since a CAR targets a single tumor associated antigen (TAA), which is not effective against tumors with heterogeneous TAA expression or emerging antigen loss variants. Further, stably engineered CAR T cells can continually and uncontrollably proliferate and activate in response to antigen, potentially causing fatal on-target off-tumor toxicity, cytokine release syndrome, or neurotoxicity without a method of control or elimination. To address these issues, our lab and others have developed various universal immune receptors (UIRs) that allow for targeting of multiple TAAs by T cells expressing a single receptor. UIRs function through the binding of an extracellular adapter domain which acts as a bridge between intracellular T cell signaling domains and a soluble tumor antigen targeting ligand (TL). The dissociation of TAA targeting and T cell signaling confers many advantages over standard CAR therapy, such as dose control of T cell effector function, the ability to simultaneously or sequentially target multiple TAAs, and control of immunologic synapse geometry. There are currently four unique UIR platform types: ADCC-mediating Fc-binding immune receptors, bispecific protein engaging immune receptors, natural binding partner immune receptors, and anti-tag CARs. These UIRs all allow for potential benefits over standard CARs, but also bring unique engineering challenges that will have to be addressed to achieve maximal efficacy and safety in the clinic. Still, UIRs present an exciting new avenue for adoptive T cell transfer therapies and could lead to their expanded use in areas which current CAR therapies have failed. Here we review the development of each UIR platform and their unique functional benefits, and detail the potential hurdles that may need to be overcome for continued clinical translation.

## Conventional CAR T Cell Therapy: Success and Challenges

Cancer immunotherapy is a rapidly growing field that has recently demonstrated clinical efficacy in the treatment of solid tumors and hematological malignancies (1–4). Numerous clinical approaches have been developed to redirect and/or augment immune function against tumor cells, including monoclonal antibodies, checkpoint inhibitors, bi-specific T cell engaging antibodies (BiTEs), and adoptive cell transfer (ACT). The application of ACT therapy for the treatment of malignant cancers has been expanded by the use of T lymphocytes engineered to express chimeric antigen receptors (CARs) (5). A CAR is a chimeric fusion protein composed of an extracellular single chain antibody variable fragment (scFv), often derived from a tumor antigen specific antibody, that is genetically fused to intracellular T cell signaling domains, thereby redirecting T cell specificity and activation toward an antigen expressed on the surface of cancer cells in an MHC-independent manner. Using optimized gene transfer technologies and advanced cell cultivation methodologies, the gene encoding a CAR construct can be efficiently integrated into the DNA of patients' non-reactive T cells, converting them into cancer antigen-reactive T cells with therapeutic potential.

Much of the clinical success of CAR T cell therapy has come in the treatment of CD19-positive B cell malignancies using CD19-specific CAR T cells (CART19) (6–9). There are currently two FDA approved CART19 products, tisagenlecleucel and axicabtagene ciloleucel. Tisagenlecleucel, developed at the University of Pennsylvania, is composed of an extracellular CD19 targeting scFv (FMC63) fused to CD137 (4-1BB) and CD3z intracellular signaling domains and has been approved for the treatment of relapse/refractory (r/r) B cell acute lymphoblastic leukemia (B-ALL) and diffuse large B-cell lymphoma (DLBCL) (10–12). Axicabtagene ciloleucel makes use of the same CD19-specific scFv but contains CD28 and CD3z as the intracellular signaling domains and was approved for the treatment of (r/r) large B cell lymphoma in October of 2017 (13). Based upon the high rates of initial cancer remission and durable responses in many patients receiving CART19 cell therapy, the ACT field has expanded with CAR T cell therapy now being applied against numerous other B cell-associated antigens with encouraging clinical response data being reported in trials targeting BCMA, CD20, and CD22 (14–16).

In spite of the unprecedented clinical success of CAR T cells in these cancer types, the use of a “living drug” has brought with it new and challenging side effects and toxicities. Upon recognition of the target tumor antigen, CAR T cell activation and expansion at a tremendous rate can result in cytokine release syndrome (CRS), a common side effect of CAR T cell therapy that is characterized by markedly elevated soluble IL2, IL6, IL10, IFNg, as well as elevated CRP, ferritin and decreased fibrinogen (8). In preclinical models, CAR-associated CRS is linked with myeloid cell release of IL-1 and IL-6, corroborating the current clinical method of CRS control with the use of the anti-IL-6 antibody tocilizumab and offering an option for IL-1 antagonists in the control of CRS (17, 18). In addition to CRS, all clinically-approved CAR T cell treatments can cause sustained B cell aplasia in patients due to the prolonged persistence and anti-B cell activity of the infused CART19 cells. While B cell aplasia as a toxicity in patients treated with CART19 cells is manageable with intravenous immunoglobulin treatment and antibiotics, severe toxicities and even death has been reported in some trials of CAR-T cell therapy where the target antigen is highly expressed on cancer cells and expressed at lower levels on normal healthy tissues, resulting in on-target, off-tumor toxicities (19–21). Based upon current practices, there are presently no mechanisms in place to quantitatively and temporally control the expansion and activation of CAR T cells following their administration.

Finally, although nearly 90% of r/r B-ALL patients achieve a complete response 1 month after administration of CART19 cell therapy, a significant number of patients still relapse (22). Only 55% of the patients that experienced an initial CR are disease-free at 1 year, but relapses are rarely observed after 1 year. Two general mechanisms of relapse occur in these patients. For some patients, relapses are associated with poor T cell function or persistence (23, 24). These are generally CD19+ relapses where the leukemic blasts maintain surface expression of the CD19 target, and the patient can accordingly be retreated with CART19. In a second set of patients, relapses occur despite a strong initial activity and engraftment of CART19 cells. In these cases, the leukemia recurs with apparent loss of CD19. Three major mechanism of CART19 tumor escape have now emerged. Multiple studies have shown that resistance to CART19 therapy is accompanied by the apparent disappearance of the target CD19 protein as a result of gene splicing, frameshifting or deletion (25, 26). In some cases, one of the two gene copies that code for CD19 on chromosome 16 is deleted, and the other copy becomes damaged as a result of mutations in coding areas of the CD19 gene, most frequently in exon 2, which encodes for the epitope recognized by the CD19 CAR. By an alternative mechanism of gene splicing, exons 2, 5, and 6 were frequently skipped in the same patients, making mutations in exon 2 largely irrelevant since the deletion of exons 5 and 6 resulted in premature termination of the CD19 protein and the deletion of exon 2 resulted in the production of a modified version of CD19, which was more stable than its standard version but not recognized by CART19 cells. Other studies have demonstrated lineage switching as another possible mechanism of CART19 resistance (27, 28). Gardner et al. reported on two unique CD19-negative relapses arising from an ALL to AML lineage switch shortly after CART19 cell therapy in two out of seven treated patients with mixed lineage leukemia (MLL) B-ALL(28). In one rare instance, the CD19 CAR gene was engineered into a single leukemic B-ALL clone (29). Here, the presence of the anti-CD19 scFv portion of the CAR and its binding to the CD19 molecule on the leukemia cell surface resulted in masked expression of CD19 target, resulting in resistance to CART19 therapy.

Thus, CAR T cell therapy that is designed to target only a single tumor antigen allows tumor cells to escape the therapy through loss of the target antigen or the antigenic epitope, with no simple and efficient method to switch the target antigen without having to make an entirely new CAR. While treatment for CD19-negative relapsed cancer after CART19 therapy may be achieved through subsequent administration of CD123 or CD22 CAR T cells, this is not without significant financial costs and safety risks (15, 30). Alternatively, T cells engineered for dual antigen specificity (e.g., CD19 and CD123) may mediate more complete remission and overcome these mechanisms of antigen escape (31).

Beyond the CART19 paradigm, the restricted targeting of a single tumor antigen by CAR T cells appears to be a major limiting factor to successful CAR T cell treatment of solid tumors. Unlike B-ALL where nearly all cancer cells express the CD19 antigen, solid tumors are often comprised of tumor cells with diverse and heterogeneous expression levels of target antigen, rendering them insensitive to CAR T cell recognition (32). This is evidenced in a trial of intravenous infusion of CAR T cells for recurrent glioblastoma (GBM) where EGFRvIII CAR T cells effectively trafficked to regions of active GBM and expression levels of EGFRvIII in the persisting lesions declined in 71.4% (5/7) of treated patients for whom post-infusion tumor was available (33). Alternative CAR strategies will be necessary to deliver multi-antigen targeting and adapt to the changes that accompany immune pressure against a single antigen.

## Universal Immune Receptors With Adaptable Specificity

CARs are architecturally rigid, modular proteins that commonly consist of an extracellular antigen targeting domain, an extracellular spacer region, a transmembrane domain, and one or more intracellular signaling domains. As such, CARs represent a forced, dominant bypass to traditional T cell receptor (TCR) binding to peptide/MHC complexes for T cell activation, but akin to the TCR, their specificity is fixed and dictated by the scFv used in the creation of the CAR construct. Thus, once the CAR is engineered into the T cell, the redirected specificity and activity of the modified T cell is permanent and not easily adapted or controlled.

**Figure 1:**
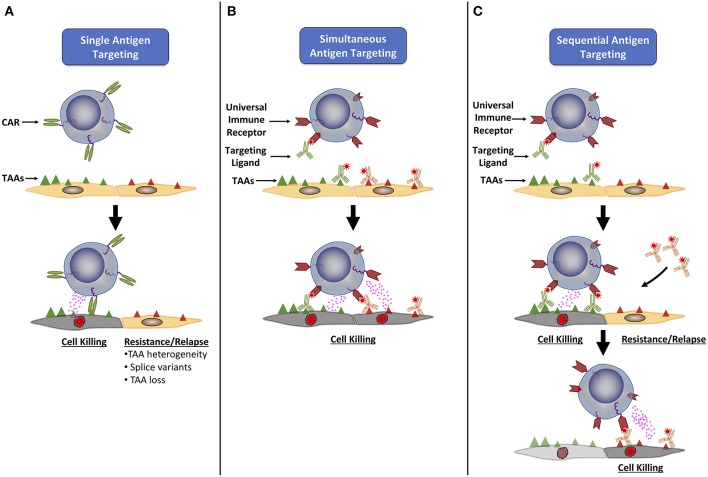
Universal immune receptors have expanded potential to target multiple tumor associated antigens**. (A)** Classical CAR T cells are only able to target a single tumor associated antigen (TAA), allowing tumor cells to evade detection through loss or down regulation of the targeted TAA, or the expression of TAA splice variants. To combat this, universal immune receptors allow for either simultaneous **(B)** or sequential **(C)** addition of ligands targeting multiple TAAs. The simultaneous targeting of multiple TAAs could lower the chance of immune evasion seen with single antigen targeting, while the ability to change the antigen target of choice over time could allow for continual targeting of an evolving tumor antigenic landscape.

In order to overcome this and other limitations of CAR T cell therapy, we and others have developed alternative chimeric receptor designs that rely in part upon the fundamental principles of conventional CAR architecture but provide the means for quantitative and temporal control of CAR T cell specificity and activity. Termed universal immune receptors (UIRs), these adaptable chimeric proteins maintain a relatively similar structure to CARs but contain an extracellular adapter domain that functions as an orthogonal bridge between intracellular T cell signaling domains and a soluble tumor antigen targeting ligand (TL). Unlike the CAR approach, this strategy allows for selective post-translational redirection of T cell specificity and function against antigen bearing tumor cells (Figure 1). Accordingly, the split structure design of UIRs has the potential benefit of being able to overcome several limitations of standard CAR therapy; namely, by allowing for dose regulation of effector function, redirection of CAR T cells against multiple target antigens or epitopes, and the ability to use a single chimeric receptor to target multiple tumor types.

**Figure 2:**
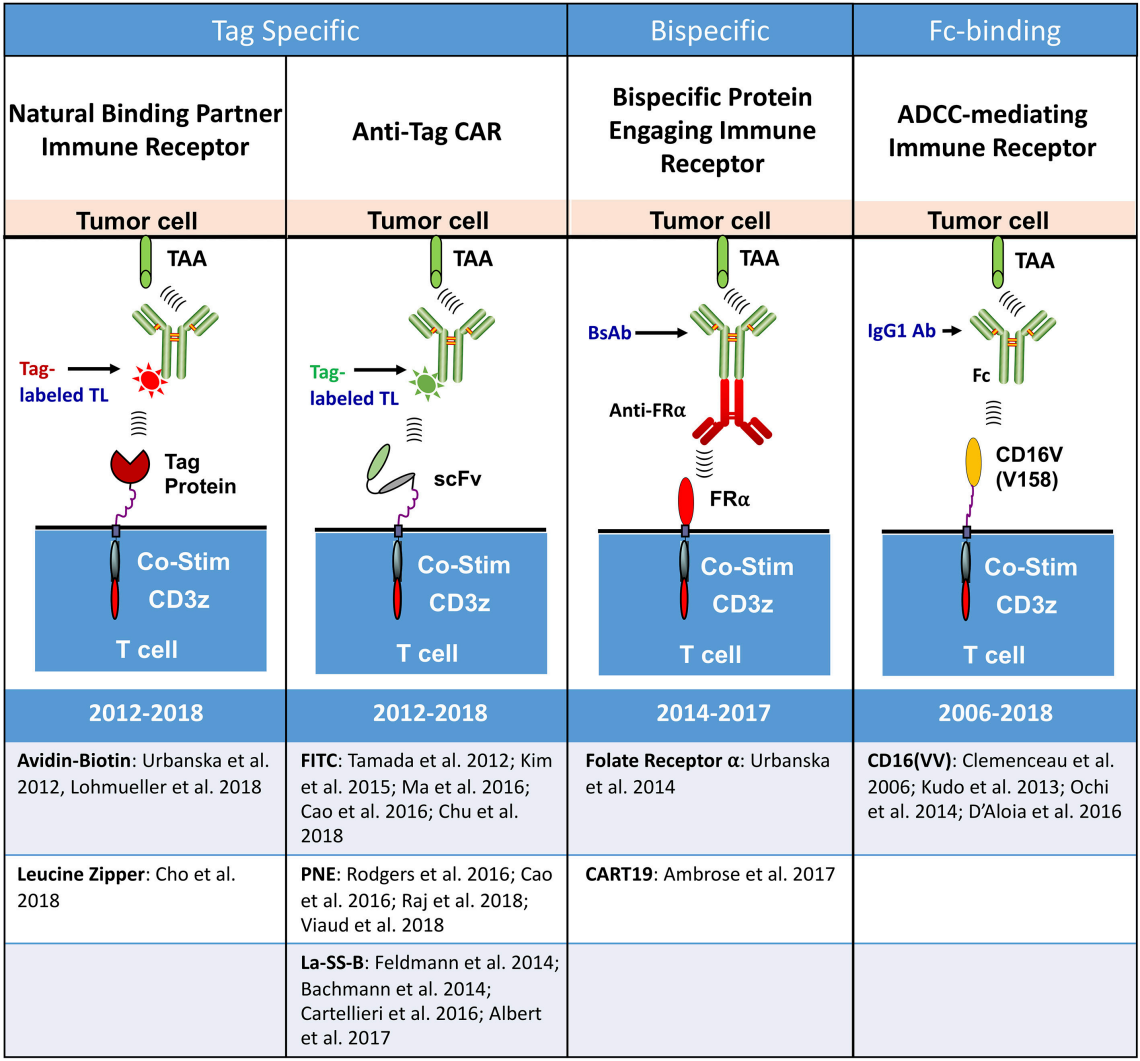
Types of universal immune receptor platforms. The bridging of intracellular T cell signaling domains and antigen targeting through an extracellular adaptor moiety is the fundamental design for all current universal immune receptors. Variations to this format have led to the development of four distinct UIR subsets. Tag specific UIRs rely on binding of the extracellular domain to a tag present on the targeting ligand. This can either be done through the use of natural binding partners, such as avidin-biotin or leucine zippers, or through binding of an anti-tag CAR to its cognate antigen tag. Bispecific protein engaging molecules underlie another subset of UIRs, engaging the T cell and tumor simultaneously to stimulate T cell effector function. The final UIR platform functions through a mechanism similar to antibody dependent cellular cytotoxicity (ADCC) by engaging tumor specific antibodies with the extracellular CD16 Fc binding domain.

Conceptually, all universal immune receptors serve the same basic function: allowing for T cell activation in response to the binding of an extracellular adapter moiety to a partnering binding domain or “tag” on an antigen-bound TL. An adapter-tag binding partnership substitutes for the standard scFv-antigen engagement which elicits T cell activation in CAR T cells. Though all UIRs fit this basic structural strategy, there are four distinct types of UIRs that have been developed to date: (i) antibody dependent cellular cytotoxicity (ADCC) receptors (34–37), (ii) Bispecific protein mediated linkage (38, 39), (iii) anti-tag CARs (40–51), and (iv) tag-specific interactions (52–54) (Figure 2).

## Fc-binding Chimeric Immune Receptors

UIRs apply many of the basic principles of ADCC to T cell therapy. In ADCC, effector cells that express Fc receptors can actively lyse a target cell, whose membrane-surface antigens have been bound by specific antibodies which serve as an immunological bridge between the Fc receptor and the target antigen. Further, clinical observations from monoclonal antibody trials suggest that ADCC mediated by FcγRIIIa (CD16)–bearing cells, such as natural killer (NK) cells, is a major mechanism of action.

In 2006, Clemenceau and colleagues reported on an early form of UIR which consisted of an extracellular CD16 domain attached to an intracellular FcIR domain (34). They noted that the gene coding FcγRIIIa displays a functional allelic dimorphism generating allotypes with either a phenylalanine (F) or a valine (V) residue at amino acid position 158. Since NK cells from donors homozygous for FcγRIIIa-158V (VV) bind more human IgG1 and IgG3 than do NK cells from donors homozygous for FcγRIIIa-158F (FF) (55), the use of CD16(VV) as an extracellular adaptor is preferred over CD16(FF) in UIR development as it allows for enhanced binding of human IgG isotypes to the receptor. Clémenceau et al. showed that human T cells engineered to express the chimeric CD16VV protein retained their natural specificity instilled by their endogenous T cell receptor, but additionally proliferated, secreted cytokine and specifically killed antigen-positive leukemia cells *in vitro* upon addition of CD20-specific (rituximab) IgG antibody. Notably, the CD16VV platform made ready use of a clinical-grade antibody without further manipulation and the activation of CD16(VV) UIR T cells was dependent upon antibody immobilization; soluble IgG, as might be found in the circulation, did not activate UIR T cells. This group later demonstrated the capacity of this system to mediate cancer regression in a preclinical model of subcutaneous human HER2+ breast cancer *in vivo* after intraperitoneal injection of HER2-specific trastuzumab and subsequent administration of CD16(VV)-engineered NK cells, NK-92^CD16^ (56). Using a similar platform, Ochi et al. showed redirected T cell specificity against HER2, CD20 and CCR4 *in vitro*, and cytotoxic effector functions against Raji lymphoma cells injected into immunodeficient mice (36). With an understanding that incorporation of a costimulatory signaling domain enhances CAR T cell proliferation, persistence and function (57–61), a CD16(VV) UIR was later developed that contained in tandem 4-1BB and CD3z intracellular signaling domains (4-1BBz), with 4-1BBz showing the greatest efficacy *in vitro* (35).

Based upon these and other findings, clinical trials using the CD16VV UIR are currently underway for the treatment of non-Hodgkin's lymphoma (CD20+), HER2-positive cancer (trastuzumab) or multiple myeloma (SEA-BCMA) (Unum: NCT02776813, NCT03189836, NCT03266692, NCT03680560). Early clinical trial results reported for the CD16(VV) drug, ACTR087 (Unum), at the low dose (0.5 × 10^6^ ACTR T cells/kg) in combination with the anti-CD20 antibody, Rituxan, included two complete responses and one partial response in 6 evaluable patients with Rituxan-resistant NHL; no T cell activation-related adverse events were observed. However, at dose level two (1.5 × 10^6^ ACTR T cells/kg) two of the nine treated patients died from serious adverse events that included severe CRS (cytokine release syndrome) and neurotoxicity^1^. Of the two events of CRS, one patient subsequently experienced a fatal case of enterococcal sepsis considered related to ACTR087 and one patient subsequently experienced a fatal case of sepsis considered not related to ACTR087. After a temporary FDA hold, these trials are again open with modified protocols and dosing.

Whether the ability of the CD16VV domain to bind to aggregate or potentially auto-reactive IgGs in the circulation or immobilized in tissues is associated in any way with these toxicities is not known, however, Fc-binding UIRs remain potentially less specific than other UIR model types due to their intrinsic ability to bind host IgGs.

## The Development of UIRs That Utilize Bispecific Targeting Ligands

The following three UIR platforms further enhance the specificity of the receptor for its TL. Bi-specific protein-binding UIRs function through co-engagement of the tumor antigen and the extracellular portion of the UIR through a soluble bispecific bridging protein. This allows for direct incorporation of co-stimulation into the T cell response, which is an advantage over current bi-specific T cell engagers (BiTEs) that only engage CD3z directly. In addition, the *ex vivo* engineering of bi-specific antibody UIRs provides an opportunity to select and expand the desired subset of T cells, whereas BiTEs can indiscriminately bind all CD3 expressing T cell, whether pro-inflammatory or immunosuppressive in function. Urbanska and colleagues developed the first bi-specific antibody UIRs using the extracellular domain of the self-protein, folate receptor α (FRα) genetically fused to CD28 and CD3z intracellular T cell signaling domains (38). In co-culture experiments, the addition of a novel bispecific antibody targeting FRα and a tumor antigen-specific antigen (CD20) led to the selective redirection of the UIR T cells against CD20+ tumor cells, while untransduced cells remained inactive. Increased secretion of IFNg, TNFa and IL-2 cytokines was dependent upon the incorporation of the CD28 signaling domain into the UIR. More recently, Aleta Biotherapeutics (Natick, MA) described a parallel technology that allows CART19 T cells to be redirected against additional tumor antigens through the use of a soluble CD19-antibody fusion protein (39). Here, the CD19 portion of the protein binds to the CART19 receptor while the scFv portion binds to the target antigen, bridging T cell and tumor cell. This technology takes advantage of the known clinical activity and persistence of CART19 cells in patients, and may provide a clinical tool to address CD19-negative relapse in CART19 recipients by redirecting the CAR T cells against CD20, using a CD19-CD20scFv fusion protein. It may also have utility in the treatment of solid tumors, however the potential induction of CRS, neurotoxicity and long term B cell aplasia in these patients remains a risk. This risk is limited in tag-specific UIR approaches in which the UIR uniquely binds to the TL and no other cell surface protein in the body.

## Tag- and Anti-Tag-Specific Universal Immune Receptors

Tag-specific UIRs exploit natural ligand-ligand binding system to facilitate receptor-TL interactions. Relying upon the known interaction between biotin and avidin, the first tag-specific UIR to be developed used dimeric chicken avidin for the extracellular domain of the receptor, thereby allowing for redirection of engineered T cells against biotinylated TLs, including both scFvs and antibodies (52). The biotin binding immune receptor (BBIR)-expressing T cells exclusively recognized, bound and killed cancer cells pretargeted with antigen-specific biotinylated antibodies, but not non-biotinylated antibodies, in a TL dose dependent manner. The versatility afforded by BBIRs also permitted sequential or simultaneous targeting of a combination of distinct antigens, allowing for tailored antigen specificity in a time and dose dependent manner. Further work on the biotin-specific platform led to the use of a high affinity monomeric streptavidin extra cellular domain (53). Cho et al. developed a tag-specific system, termed SUPRA CAR, using synthetic and human derived leucine zipper domains with the unique ability to control effector function through affinity tuning of the UIR-TL interaction (54).

Anti-tag CARs are UIRs that use a standard scFv-based CAR receptor whose cognate antigen is a small peptide or molecule tag. These tags are genetically fused or chemically conjugated to various TL types, ranging from small molecules to antibodies. These tag-labeled TLs serve as a bridge between the anti-tag CAR T cell and antigen expressing tumor cell, leading to titratable elicitation of effector function. Anti-tag CARs have been designed to bind FITC (40, 43, 44, 47, 50), a peptide neo-epitope (PNE) from the yeast transcription factor GCN4 (45, 47, 49, 51), and E5B9 peptide derived from nuclear antigen-La-SS-B (41, 42, 46–48). The inherent flexibility of these tag-based systems may allow for refined engineering of either the receptor or the TL to optimize T cell effector function against specific tumor types. Though none of the anti-tag CARs are currently used in the clinic, both Endocyte^2^ (anti-FIT CAR) and Calibr^3^ (anti-PNE CAR) are progressing toward clinical trial testing of their respective platforms.

There are many facets of these platforms that will need to be precisely designed in order for their true potential to be reached in the clinic, but the current UIR literature clearly illustrates the significant promise that these receptors hold for clinical use.

## Aspects of Universal Immune Receptor Design

Due to the split nature of the receptor and TL, careful consideration needs to be given to the engineering of each member and portion to ensure optimal functionality against targeted tumors. UIR composition will likely be impacted by the same factors that affect standard CAR design, including selection of optimal stimulation and costimulation domains, hinges, transmembrane regions, and targeting domains (5). CAR T cells typically use scFvs as their standard targeting moiety, though other molecules such as DARPins and cell adhesion proteins have been used as well (62, 63). Each of these unique TLs must be engineered with respect to the receptor construct itself. A broader variety of TL molecules have been used to redirect UIR T cells, including antibodies, scFvs, bispecific scFvs, Fab fragments, nanobodies, and small molecules (43, 45, 46, 52). The expanded repertoire of potential TL types brings with it the added ability to alter many properties of the UIR system itself, such as affinity of the receptor for the TL, affinity of the TL for the target antigen, epitope binding valency, pharmacokinetics of effector function, and tumor penetrance and distribution of the TL (48, 54, 64, 65). Tag valency and placement are also key considerations for TL development, with the potential to impact effector function and induce target-independent activation (45, 66). Emerging data suggest that these factors will have a major impact on the design of optimally functional UIR systems against each target antigen.

## Controlling the Immunologic Synapse

One key aspect of CAR and BiTE design is the optimization of the immunologic synapse formed between the T cell and target cell (67). Hinge domains, typically derived from IgG4 or CD8 molecules, serve to extend the scFv farther from the plasma membrane for greater efficiency in ligand binding and tumor lysis (5). The length of the hinge region can have a strong impact on CAR function, with optimal spacer length varying based on the epitope being targeted (68, 69). Insertion of a flexible hinge region may also relieve steric inhibition between CAR binding moieties and cancer epitopes, as seen in the improved lysis capabilities of a MUC1-targeted CAR (70). Thus, the extracellular spacer domain in standard CARs likely needs to be optimized for each specific antigen target to allow for maximum CAR T cell efficacy.

The spatial relationship between a UIR bearing T cell and a tumor cell can be altered through either the extracellular spacer domain of the receptor, the size of the TL, or the placement of the tag on the TL. Using site-specific tag conjugation and molecular engineering techniques, anti-tag UIR studies show that tag placement on the TL is important for optimizing its efficacy against each target antigen and is impacted by whether the epitope is distal or proximal to the tumor cell membrane (44, 45). For instance, one study demonstrated that targeted placement of the tag proximal to the epitope binding site increased the antitumor activity in the targeting of CD19+ tumor cells (45). Additional studies directly compared site-specific tag conjugation to random tag conjugation and noted that random incorporation led to a decrease in efficacy both *in vitro* and *in vivo* (44). Taken together, these results demonstrated that tag placement can be optimized independently from the receptor through the use of site-specific tag conjugation of the tag to the TL.

Changing the extracellular spacer of the UIR is another method for synapse space optimization. Using their PNE tag system, Rodgers et al. showed that the use of a short IgG4 hinge region leads to increased efficacy in the targeting of CD19+ cells (45). In additional experiments targeting CD20 and CD22 with UIR T cells, the importance of pairing the correct hinge with tag placement for optimal tumor cell lysis was further demonstrated (44, 45). Taken together, these results underscore the need for precise engineering to optimize UIR T cell activity for a given antigen. Unlike with standard CARs this optimization can be done through alterations to the TL, meaning that a single UIR receptor could still be used to optimally target multiple antigens. Since exogenous development of TLs can be performed independent of manipulation of the receptor itself, this could potentially allow for higher throughput screening and optimization of effector function compared to optimization techniques used for individual CARs.

## Toxicities and Methods of Regulating Effector Cell Function

As mentioned earlier, CAR T cell therapy is not without toxicity. On-target, off-tumor T cell engagement is a potentially fatal side effect of CAR therapy. This is especially troublesome in the targeting of solid tumors, which often overexpress antigens already present on normal tissue. Targeting of the commonly overexpressed tumor antigen HER2 was fatal in a single patient clinical trial, with complications potentially arising from recognition of low-level HER2 expressed on normal lung cells (20). An early clinical trial for metastatic renal cell carcinoma was forced to cease treatment after four of the eight patients exhibited abnormalities in liver enzymes, likely due to targeting of carbonic anhydrase IX (CAIX) on bile duct epithelial cells by the infused CAIX-specific CAR-T cells (21). Additionally, cytokine release syndrome, caused by robust T cell activation post-infusion, can cause serious health complications in patients (71, 72). Neurological toxicity, including delirium in fevered states and global encephalopathy, has also been reported in relation to CD19-directed CAR T cell therapy and may in part be linked to CRS (73, 74). Though CRS is currently managed by infusion of the anti-IL6 antibody tocilizumab, the ability to dose-control CAR T cell effector function could alleviate its need.

Improving the clinical safety of CAR T cells while retaining antitumor function is currently a major area of research. Significant effort is being made to develop effective safety mechanisms to shut off, or otherwise titer, CAR-T cell function in the event of unmanageable toxicity. These “switch” functions use exogenous molecules to induce either the activation or death of CAR-T cells. The first suicide switch system transferred the herpes simplex virus thymidine kinase (HSV-TK) gene into donor T cells. Upon administration of ganciclovir, the thymidine kinase of HSV-TK catalyzes the transformation of ganciclovir to the lethal triphosphate ganciclovir (GCV) (75). Although found to be safe and efficacious clinically (76), the HSV-TK system has drawbacks in its potential for immunogenicity (77). Another example of an “off-switch” is the inducible Caspase 9 system (iCAsp9) which causes apoptosis in activated T cells upon administration of the small molecule AP1903. Upon treatment, those cells which express the iCasp9 transgene are rapidly and preferentially killed, allowing for cessation of T cell activity *in vivo* (78, 79). The iCasp9 CART system is currently under clinical investigation, targeting advanced melanoma, neuroblastoma, sarcoma, and other solid tumors which express GD2 (80). The Casp9 gene has also been fused to rapamycin binding domain FKBP12, allowing for use of rapamycin as a kill-switch molecule (81).

ADCC mediated depletion of T cells is another possible method of elimination. Expression of the EGFR extra cellular domain in T cells allows for depletion through infusion of cetuximab and has been used in clinical trials testing MUC16 targeting CAR T cells (82, 83). A similar methodology was developed by expressing CD20 on T cells, therefore enabling rituximab infusion as a means to deplete engineered T cells in mouse models (84). This system, named RQR8, is currently being used in clinical trials for non-Hodgkin lymphoma and multiple myeloma (NCT03590574, NCT03287804). A study comparing iCasp9, HSV-TK, and CD20 depletion suicide switches concluded that both the iCasp9 and CD20 allowed for rapid and effective T cell depletion, albeit iCas9 appeared more efficient (85). Clinical trials have also been conducted using mRNA electroporated CAR T cells, which gradually lose CAR expression over time as the mRNA degrades (86).

As an alternative approach to decrease off-tumor cell killing, antigen-specific inhibitory chimeric antigen receptors (iCARs) have been developed that comprise an extracellular antigen-binding domain and an intracellular CTLA-4 or PD-1 signaling domain. This allows iCAR T cells to transmit inhibitory signals into the T cell to suppress the T cell response only upon binding to a self-antigen expressed on normal cells. In this way, strong therapeutic function may be retained against target tumor cells, which lack the healthy tissue antigen, while any healthy cell carrying the self-antigen would trigger CTLA-4 or PD-1 signaling and be preferentially spared (87). Similar to iCARs, masked CARs (mCARs) remain masked from antigen recognition until a protease which is commonly activated in the tumor microenvironment unmasks the antigen recognition domain of the CAR, thus allowing for localized tumor recognition and activity. As proof of concept, a third generation mCAR specific for EGFR was “masked” using an N-terminal peptide which blocks the antibody binding site of an EGFR-specific CAR (88). Upon exposure to tumor-associated proteases, N-terminal peptide was cleaved and the activity of mCAR T cells against EGFR enhanced.

Unlike these approaches, UIRs utilize methods of effector function control that do not involve the direct genetic engineering of a “kill-switch” or other genetic modification to the T cell genome. In order for UIRs to engage antigen expressing tumor cells, TL must be introduced to the system; in the absence of TL the UIR T cells are viable but inactive. This is not the case for suicide switch systems which result in the deletion of the engineered T cell product and thus a termination of the therapy. UIR T cells persist in the circulation of treated mice following the clearance of tumor and the discontinuation of TL administration (44, 45). Accordingly, discontinuation of TL administration can prevent adverse effects associated with the persistent CAR T cell activity while also providing the opportunity for subsequent TL administration upon cancer relapse.

**Figure 3:**
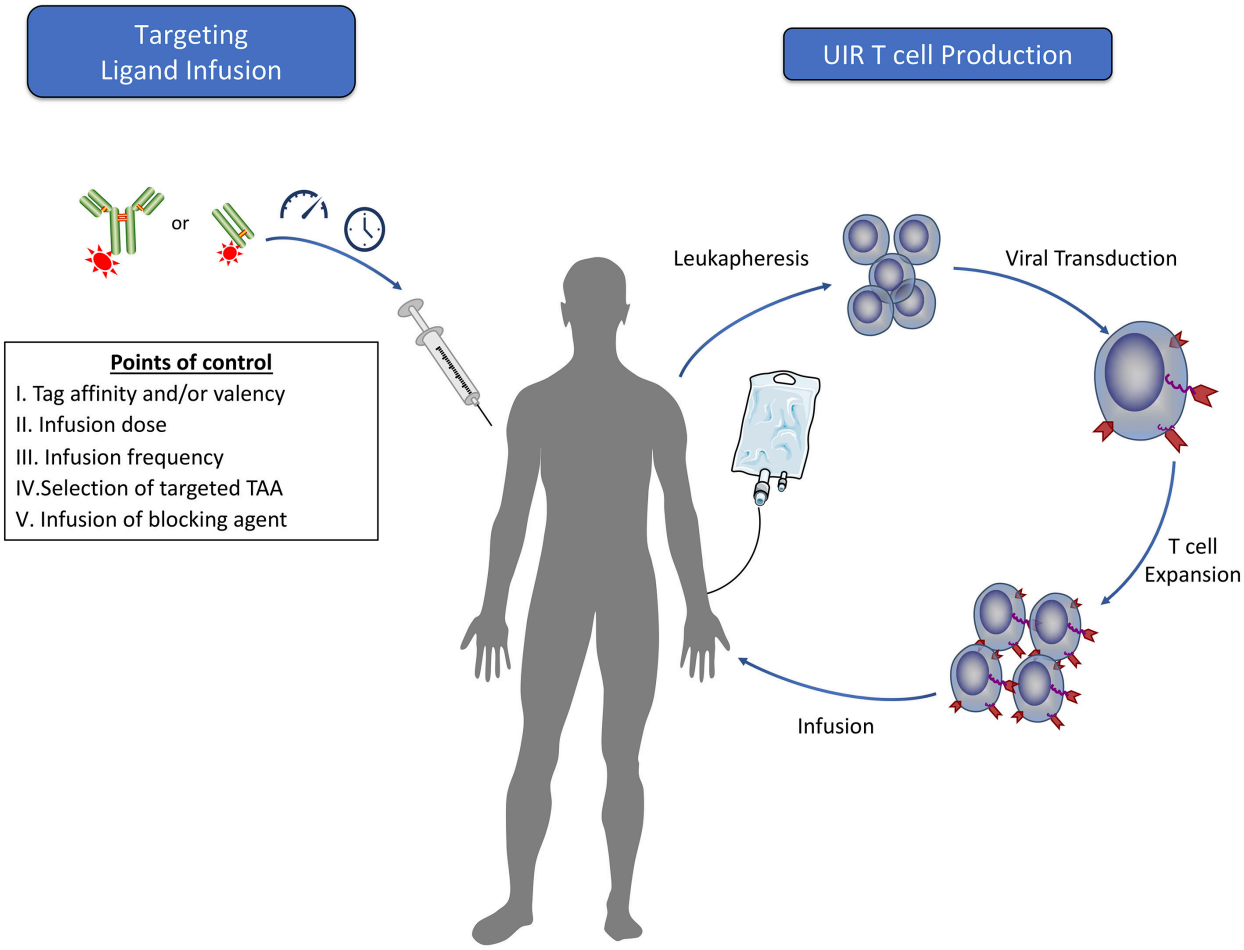
Clinical steps for adoptive T cell therapy using universal immune receptors. The production of universal immune receptor T cells for clinical use would follow methods similar to those currently used for classical CAR T cells. Many of the potential benefits that a UIR system may confer would rely upon the dosing of targeting ligand into the patient. Prior to infusion, engineering of targeting ligands can be done independently of the receptor to maximize effector function against each target antigen. The ability to alter targeting ligand dose amounts and frequency would allow for tight regulation of T cell effector function and serve as a way to control T cell function to mitigate potential toxicity while achieving cancer regression. In the case of acute toxicity, infusion of a blocking agent may provide rapid cessation of T cell effector function. Furthermore, infusion of targeting ligands against multiple tumor antigens could offer the added benefit of targeting a heterogeneous cell population in the tumor with a single T cell product.

In addition to the benefits of allowing for safe, engineered T cell persistence, UIR systems provide a mechanism for quantitative control of effector cell function through manipulation of the administered dose or alterations to the dosing schedule (Figure 3). In a manufacturing and infusion approach similar to that used for CAR T cells, patient T cells that are *ex vivo* engineered to express the UIR can be administered prior to, simultaneously with, or after TL dosing. As described below, these TL dosing regimens allow for exquisite control of cytokine secretion and tumor lysis by UIR T cells *in vitro* and *in vivo*, and dose escalation over the course of a treatment can combat relapse in mouse models (44, 45, 52, 54). These aspects of UIRs have significant clinical implications.

Nearly all patients with B-ALL that receive highly active CART19 T cells experience some level of CRS ranging from mild flu-like to life threatening symptoms (89). Here the infusion of CART19 cells results in a dramatic inflammatory process associated with supraphysiological T cell proliferation and significant cytokine elevations and is often associated with burden of disease. Since dose titration of TL allows for controllable cytokine secretion by UIR T cells *in vitro* and *in vivo* (45), a reduction in single TL dosing, the application of metronomic dosing or the use of an escalating TL dosing regimen would be anticipated to reduce the incidence or severity of CRS in the clinic. In a Nalm-6 model of UIR T cell treated leukemia, low dose administration of a CD19-specific TL induced low serum cytokine levels while triggering tumor clearance that was markedly slower than, but ultimately as effective as, that achieved with conventional CART19 cells (45). In some mice treated with UIR T cells, the Nalm-6 tumor later relapsed, at which time these mice were retreated with a higher dose of the TL, resulting in a secondary antitumor response that was similar to CART19 cells. This establishes the principle that UIR T cells can be administered in combination with an initially low but escalating TL doses to restrain CRS yet still achieve potent antitumor activity that is comparable to conventional CART19 cells.

In addition to CRS, patients receiving CD19-targeted T-cell therapy have prolonged B cell aplasia. Although this is a manageable toxicity it requires the need for potentially life long intravenous IgG (IV/IG) “replacement dosing” and antibiotics as a supplement (19). As demonstrated using a UIR syngeneic CD19+ leukemia model, cessation of anti-CD19 TL dosing after tumor clearance allows for the reestablishment of the endogenous B cell population, even in the continued presence of inactive UIR T cells, and is therefore an important step toward improving the quality of life for patients post-treatment (44).

Beyond TL dosing regimens, altering the affinity between the UIR and its cognate tag, or the affinity between the TL and the target antigen, are also feasible methods for enhanced control of the induced level of T cell effector function (35, 54). In the SUPRA CAR system, which relies upon synthetic leucine zipper pairing between the UIR and TL, affinity regulated function was demonstrated both *in vitro* and *in vivo* through alteration of the leucine zipper pairing affinity (54). This ability to control the affinity of TLs within a UIR system may provide an additional layer of safety, analogous to the use of lower affinity scFvs in CARs to provide specific targeting of tumor cells that express high levels of antigen, but not normal tissue expressing low level antigen (90, 91).

In a similar fashion, the valence of tags per TL molecule also impacts the efficacy of UIR T cells. In one example, CD19 and CD22 Fabs with bivalent, site-specific placement of FITC consistently show greater potency *in vitro* and *in vivo*, compared to monovalent forms (44). However, the impact of tag valence has not been observed across all UIRs. The PNE system showed mixed results of TL potency when comparing site-specific monovalent vs. bivalent tag placement. Treatment of Nalm-6 xenografts with anti-CD19 Fabs and PNE CAR T cells showed equivalent efficacy between mono- and bivalent TLs, with a minor increase in T cell expansion seen in the monovalent group (45). It was also noted that optimal tag valency may be impacted by the selected hinge region of the UIR, since shorter hinge domains may sterically limit the ability of two independent UIR receptors to bind a single multivalent TL.

As a more direct means of T cell control, UIR T cell effector function can also be halted through the specific, timed blockade of the UIR-TL interaction using specific UIR or tag binding competitors (40, 54) (Figure 3). This method may be more rapid in effectiveness than withdrawal of targeting agent alone, which would depend upon the *in vivo* clearance kinetics of the TL to halt T cell function. *In vivo* studies with both the anti-FITC UIR and SUPRA CAR systems have shown that administration of an inert non-targeting agent containing the necessary UIR binding moiety in excess is a safe and effective method of inhibiting both cytokine secretion and tumor clearance (40, 54). This again has the added benefit of allowing the UIR T cells to persist in an inactive form after the delivery of blockade therapy, compared to suicide switch systems which result in T cell deletion and thus termination of the therapy.

## Flexible Antigen Targeting

Translating the success of CAR T cell therapy achieved in hematological malignancies to the treatment of solid tumors has been largely unsuccessful but remains a significant clinical opportunity (2). One key factor for these unfavorable results is that solid tumors tend to have heterogeneous cell composition and therefore lack ubiquitous expression of a single tumor associated antigen (TAA) (32). Even when responses appear initially complete, antigen loss is another major challenge which CAR T cell therapy must overcome.

In clinical CART19 trials, tumor cell evasion can occur through the loss of antigen expression or the emergence of CD19 splice variants that lack the target epitope (26, 28, 92). By virtue of their design, CAR-T cells target only a single tumor antigen. Although a second epitope could theoretically be targeted using a second infusion of CAR T cells, this would require expensive and time-consuming production of a new T cell product for each patient. Use of dual targeting CARs (93–95), T cell products containing two distinct CAR populations (31), or CAR T cells transduced to express two independent receptors (96) are all feasible approaches to overcome this hurdle, but these methods still restrict the total number of targetable antigens and do not address potential toxicity issues.

One of the main benefits of UIRs is their ability to target multiple antigens either sequentially or simultaneously (52) (Figure 1). Simultaneous targeting may alleviate the risk of antigen escape variants during treatment, while sequential targeting would allow for the use of the same engineered T cell to be redirected against additional antigens that arise as the tumor evolves through infusion of new TLs. Biopsy and proteomic characterization of a patient's relapsed/refractory tumor could also inform the tailored dosing of a sequential TL(s) at later treatment time points, a property unique to UIR platforms (49). An array of various solid and hematologic target antigens have already been successfully targeted by current UIR platforms in preclinical models, showing the expanded lytic potential that these systems have (34, 35, 42, 43, 45, 52, 54). Urbanska et al. demonstrated that the same UIR T cell product could be readily used to target either mesothelin, FRα or EpCAM (52). A study by Cartellieri et al, also demonstrated that the use of two monospecific TLs or a single bispecific TL led to efficient redirected T cell activity against CD33+ CD123+ AML cells (46). Interestingly, the bispecific TL had enhanced anti-tumor efficacy when compared to the addition of its two individual monospecific subparts.

Beyond targeting multiple antigens on the cancer cell surface, simultaneous targeting of both tumor and non-tumor antigens has the potential to further enhance therapeutic effectiveness. Multiple studies have shown delayed tumor growth and increased T cell infiltration through targeting of antigens expressed on the tumor vasculature (97–99). Synergistic effects were observed with the combined targeting of tumor vasculature antigen VEGF receptor-2 and multiple tumor specific antigens (99). Similarly, targeting normal but tumor-promoting stromal cells using CAR T cells specific for fibroblast activating protein can inhibit tumor progression (100). Targeting of immunosuppressive cells in the tumor microenvironment, such as M2-like macrophages, may also enhance T cell function (101). While these are yet untested approaches, the potential to combine targeting of tumor and non-tumor antigens with a single cell product could provide synergy and benefit to overall efficacy of treatment, and would be an approach unique to UIR T cells.

## Manipulating T Cell Memory, Differentiation, and Individual Subsets

Most current clinical grade CAR T cell products are made from a patient's polyclonal T cell product without additional sorting for T cell subpopulations. It is well documented that certain T cell subtypes, such as naive and central memory T cells, have enhanced proliferation and persistence (102), and may therefore represent better starter cell subsets for use in engineered T cell therapy. Indeed, increased frequencies of CD4+ and central memory T cells in the pre-infusion product correlates with enhanced T cell persistence post-infusion, and the development and maintenance of CAR T cells that skew toward a central memory phenotype is associated with increased efficacy in patients on various CAR trials, including in B-ALL patients with sustained remissions (7, 9, 103, 104). Thus, identifying approaches that promote these favorable T cell properties is likely to improve clinical efficacy of the UIR approach. In this line of study, Rodgers et al. found that TL dosing can impact UIR T cell differentiation (45). Here the ability to titrate the effector activity of PNE-specific UIR T cells was shown to impact the post-infusion differentiation of the T cells *in vivo* using immunodeficient mouse models. Specifically, a lower dose of a CD19 targeting TL achieved the highest frequencies of CD4 and CD8 T cells with a CD45RA–CD62L+ central memory phenotype and a lower frequency of terminal effector cells in comparison to the high dose TL condition or to standard CART19 cells.

More recently, Viaud and colleagues used a murine form of the PNE–specific UIR system to examine the impact of the TL dose schedule on the formation of UIR T cell memory in a syngeneic mouse model of CD19 targeted therapy (51). In the immunocompetent syngeneic model, CD19+ B cell reconstitution after CD19 targeted therapy provides a potential depot for chronic antigen stimulation. The investigators found that both the timing and dosage of the administered CD19-specific TL regulated the amplitude and the phenotypic distribution of UIR T cell expansion. Notably, acute introductory TL dosing followed by a 3 week “rest” and a subsequent acute second TL cycle precipitated a dramatic T cell expansion after the second TL cycle, while longer TL dosing cycles with a reduction in the rest period stymied T cell expansion. The benefits of acute TL dosing and a rest phase was observed at both high and low TL dose concentrations. At the point of robust T cell expansion, TL expanded T cells predominantly displayed a CD8 effector and effector memory phenotype. Following T cell contraction, however, there was a relative reduction of these effector subtypes and a parallel retention of long-lived central memory cells, even in the absence of continued TL dosing. These findings demonstrate that the rest phase between TL dosing cycles is vital to both UIR T cell expansion and the formation of T cell central memory, and that the duration of the rest phase between TL cycles is more important than the TL dosing concentration.

While many UIRs function through a single tag binding domain, Cho et al. developed orthogonal UIRs which function through the binding of specific leucine zipper proteins on the receptor and TL (54). Since distinct pairs of leucine zippers have exclusive interactions with one another, they can be used to expand control of T cell function through split signaling domains and/or activation of specific T cell subsets. The use of split signaling domain CARs was developed to enhance safety, necessitating the need for dual antigen recognition by a first generation CAR and a chimeric costimulatory receptor (CCR) to elicit and focus robust effector function against tumor cells (105, 106). Split signaling functionality in a UIR system would allow for even tighter “AND” gate control of T cell function, with the added ability to independently dose-titrate the activity of each split receptor and modulation of targeted antigens with a single cell product (54). Control of individual T cell subsets is also achievable with orthogonal UIRs, allowing for incorporation of unique signaling domains into distinct cell subtypes and external control of signaling pathways through administration of specific TLs. With further development these logic gating UIRs have the capability of expanding the level of precision and tuning of immune functionality in T cell therapy.

## Potential Issues and Challenges for UIRs

Though there are many benefits to developing UIR platforms for clinical use, they come with their own unique set of potential challenges. At the forefront is the potential for immunogenicity. Immunogenicity in CAR T cell treatment has been documented previously, most commonly in the development of human anti-mouse IgG antibodies (HAMAs) in response to murine derived scFvs (86). One instance of HAMA induction led to anaphylaxis and cardiac arrest in a patient receiving multiple and delayed infusion of an anti-mesothelin CAR bearing a murine derived scFv (107). Most UIR systems at present rely on the use of molecules that may elicit a similar immune response in patients. Though some UIR platforms claim to have low likelihood for immunogenicity (41, 42, 45, 46, 48), careful clinical evaluation will be required for each UIR, especially those that uses non-endogenous proteins in their design.

The selection of optimally designed TLs also comes with its own unique challenges. To date, the CD16VV UIR is the only current platform to use unmanipulated clinical grade reagents as TLs. Other platforms, such as the FITC UIR, have used clinical grade reagents such as trastuzumab and cetuximab randomly conjugated with FITC molecules (40). Though these reagents were functional *in vitro* and *in vivo*, the addition of randomly conjugated molecules may lead to batch variation during TL production. The use of novel site-specific conjugation methods could mitigate this issue and allow for more consistent TL production using clinical reagents. All other platforms rely on the use of clinically untested TLs. Each system might then have to demonstrate clinical safety of the TL independently of the UIR T cells, which could come at a significant cost. Furthermore, the clear importance of tag placement on UIR effector function may necessitate the development of TLs genetically engineered to either contain the tag itself or contain introduced conjugation sites that subsequently allow for site-specific tag conjugation. This eliminates the use of clinically validated reagents and may further increase cost on TL production.

T cell expansion and persistence may be another issue with UIR T cells, as these two factors are key correlatives for predicting durable clinical remission (10, 24, 108, 109). Establishment of long-lived CART19 cells has been observed in patients years after initial infusion (11). Persistence of CAR T cells in the treatment of solid tumors has become a major point of focus in the field, though the reasons for poor persistence in solid tumor treatment are unclear and potentially multifactorial (110–112). Nevertheless, persistence of CAR T cells may be aided in part by continual antigen stimulation, as it is hypothesized that CART19 cells as a result of continual stimulation of the CAR T cells by reconstituting CD19+ B cells (9). While the ability to withdraw TL from UIR treatment might mitigate toxicities of CRS, neurotoxicity, and B cell aplasia, continual TL dosing and availability of antigen may be needed to induced T cell activation, expansion, and long term persistence. In preclinical models, UIR T cells have the capacity to persist in the absence of TL for several weeks, and can subsequently be reactivated with secondary TL dosing (45). The degree to which UIR T cell persistence is achievable in patients is not known, however optimization of TL dosing amount and frequency may be needed for each individual tumor antigen in order to achieve maximal T cell expansion and persistence.

T cell infiltration into solid tumors is associated with good clinical outcomes in some cancers (113). T cells can face difficulties penetrating the tumor stroma, accumulating around the periphery of the tumor mass or becoming excluded entirely (114). For UIR T cells, TLs will need to be able to penetrate and retain in the tumor to facilitate UIR engagement with tumor antigens. Akin to antibodies and their derivatives, TL penetrance will likely be impacted by multiple factors, including TL size, affinity for its cognate TAA, and biological half-life. A balance between molecular mass and binding affinity of TLs has a direct effect on tumor penetrance for both antibodies and scFv (115). As such, fine tuning these properties should be considered and may be necessary for each new TL being developed for antigen targeting.

Finally, one of the most unique aspects of UIRs is their ability to target multiple tumor antigens. In an ideal setting, simultaneous targeting of multiple antigens has the potential to broaden the initial antitumor response and mitigate antigen escape, while the ability to sequentially target multiple antigens could serve as a method to adapt T cell effector specificity and function to inter- and intratumoral changes that may occur over the course of treatment. Multi-antigen targeting functions only if multiple TAAs have been discovered and deemed safe, with cell lineage specific molecules such as CD19, CD20, CD22, and BCMA appearing to be the best druggable targets at present, although GD2 and EGFRvIII hold significant promise. Though much effort is being put into the discovery and testing of CAR T cells against new TAAs, it is a long and arduous process to discover a single safe and effective target (116). Though UIRs already provide several beneficial features over CAR T cells in the targeting of single TAAs, the full utility of UIRs will be reached when the cadre of TAAs is expanded.

## Conclusion

Genetic engineering of T cells to target and eradicate tumors has been met with unprecedented clinical success in the treatment of hematologic malignancies. Recent FDA approval of CAR T cell therapies for the treatment of CD19+ malignancies has firmly established the use of adoptive T cell therapy as a viable clinical option for the treatment of disease. The identification of new target antigens coupled with improved CAR T cell design and manufacturing techniques are staged to overcome the hurdles that currently face expanding CAR T cell use across more cancer types.

Universal immune receptors are a part of this evolution, and offer the potential to improve upon many of the pitfalls that accompany CAR T cell therapy. The ability to target multiple antigens with a single, standardized immune receptor via the use of exogenous, inert targeting moieties represents an expanded approach to tumor eradication without the necessity to re-engineer or develop unique receptors for each target. Engineering of the immunological synapse is also possible without having to redesign the entire chimeric immune receptor itself, but rather through precision modifications to the TLs. Combined, these factors have the potential to greatly reduce manufacturing costs and increase development speed, especially in cases where multi-antigen targeting is beneficial to patient outcomes. UIRs also function to limit the risk of potentially fatal toxicities that may limit the expanded use of standard CAR T cells. The necessity for scheduled infusions of TL grants temporal and quantitative control over both T cell effector function and cytokine release. Pharmacokinetics and pharmacodynamics can be titrated in a more precise fashion than current achievable for conventional CAR T cells, thereby limiting the possibility of aberrant T cell activity without a means of control.

Finally, UIRs can be combined with other advancements in the field of adoptive T cell therapy to generate a true universal T cell product. Allogeneic CAR T cells lacking endogenous TCR and CD52 expression can evade detection and eradication by the unmatched recipient's immune system, resulting in a universal donor T cell product for potential widespread clinical use (117). Combining these universal T cells with a UIR creates the opportunity to significantly reduce the cost burden associated with the personalized medicine manufacturing approach of current CAR T cells. These cells could then be engineered with further enhancing elements to provide an optimized universal T cell therapy (104, 118, 119).

Much work remains to be done in translating UIRs to the clinic, however progress in the field has occurred rapidly over the past few years with growing industry support. With continued pursuit of optimal UIR engineering, these receptors could one day represent the keystone around which the next generation of adoptive T cell therapy is created.

## Author Contributions

NM and EH contributed to the literature review and drafting of the manuscript. DP contributed to the conception, drafting, and critical revision of the manuscript.

### Conflict of Interest Statement

The authors declare that the research was conducted in the absence of any commercial or financial relationships that could be construed as a potential conflict of interest.

## Footnotes

^1^https://www.sec.gov/Archives/edgar/data/1622229/000119312518068918/d416842ds1.htm.

^2^https://endocyte.com/pipeline/

^3^https://www.scripps.edu/news-and-events/press-room/2018/20180625-calibr-abbvie.html
